# Anatomographic Variants of Sphenoid Sinus in Ethiopian Population

**DOI:** 10.3390/diagnostics10110970

**Published:** 2020-11-19

**Authors:** Tizita K. Degaga, Abay M. Zenebe, Amenu T. Wirtu, Tequam D. Woldehawariat, Seife T. Dellie, Jickssa M. Gemechu

**Affiliations:** 1 Department of Biomedical Sciences, Menelik II Health Science College, Kotebe Metropolitan University, Addis Ababa 31228, Ethiopia; kinfetizita@gmail.com; 2 Department of Anatomy, College of Health Sciences, Addis Ababa University, Addis Ababa 9086, Ethiopia; abaymulu@gmail.com (A.M.Z.); amannut2002@gmail.com (A.T.W.); 3 Department of Radiology, College of Health Sciences, Addis Ababa University, Addis Ababa 9086, Ethiopia; tequamrad2018@gmail.com (T.D.W.); seifeteferi@gmail.com (S.T.D.); 4 Department of Foundational Medical Studies, Oakland University William Beaumont School of Medicine, Detroit, MI 48309, USA

**Keywords:** anatomographical variation, sphenoid sinus, septation, pneumatization, Ethiopian population

## Abstract

Neurosurgeons often neglect the sphenoid sinus due to its deep location and difficulties in accessing during surgical interventions. Disease of the sphenoid sinus is difficult to diagnose since its presenting symptoms are difficult to recognize. Moreover, compared with other paranasal sinuses, the sphenoid sinus is considered the most variable air sinus in terms of its degree of pneumatization, number and position of inter-sinus septa, and its relationship with the surrounding anatomical structures. Anatomical variations of the sphenoid sinus are significant from a neurosurgical point of view. Understanding of these variations and its relationships with surrounding structures such as the internal carotid artery, optic nerve, and pituitary gland are clinically relevant to minimize injuries associated with surgical procedures that involve sphenoid sinus. We implemented principles of imaging using computed tomography to elucidate any anatomical variations of the sphenoid sinus in the Ethiopian population. We conducted a prospective study in 200 patients with ages 18–79, who underwent scans of the sphenoid sinus at the Tikur Anbessa Referral Teaching Hospital in 2017–2018. Our findings revealed an incidence of anatomographical variations in terms of pneumatization that varied between 2–50%. These variants include 2% conchal, 25.5% presellar, 50% sellar, and 22.5% postsellar pneumatization. We also demonstrated anatomographic variants in terms of septation, 77.5% single complete septa, 11.5% single incomplete, 10% double septa, and 1% absence of septa. In summary, the sellar pneumatization was found to be the most clinically relevant anatomographic variant among Ethiopians participating in the study, of which 90% were tomographically single septated. These variants must be taken into consideration during trans-sphenoidal surgery and knowledge of the variations has clinical implication in minimizing injuries during invasive surgical procedures involving the sphenoid sinus.

## 1. Introduction

The sphenoid sinus is the most inaccessible paranasal sinus, enclosed within the body of the sphenoid bone and intimately related to numerous neurovascular and glandular structures [1]. The failure of surgeons to understand the racial variations of the anatomical landmarks of the sphenoid sinus is often described as a potential risk factor in clinical interventions [2]. The presence of Onodi cells may be accompanied by morphological variations of the neighboring anatomical structures [3]. Such anatomographic variants carry significant surgical implications in sinonasal regions.

Computerized tomography (CT) is an imaging modality used for diagnosing diseases and evaluating injuries. It also plays an important role in the diagnosis of anatomical variations, which has relevant implications in clinical decision making during surgical interventions. CT of the paranasal sinuses reveals a wide spectrum of findings associated with the normal pneumatization processes inside the sinus cavities and in the adjacent marrow spaces [4]. The sphenoid sinus received clinical significance after neurosurgeons discovered the trans-sphenoid approach for pituitary tumor surgeries in the new era of minimal invasive surgery [5]. These mucous membrane lined air cells are situated within the body of the sphenoid bone, communicating with the roof of the nasal cavity through an opening into the sphenoethmoidal recess. It is closely related with the surrounding vital structures such as optic chiasm, cavernous sinus, pituitary gland, and internal carotid arteries [2,6].

When compared to other paranasal sinuses, the sphenoid sinus follows a different developmental pattern [7]. Its formation takes place in the body of sphenoid bone, beginning as an invagination of the nasal mucosa into the posterior portion of the cartilaginous nasal capsule between the third and fourth months of fetal life. A recess appears at birth, which is present between the presphenoid body and the sphenoid concha. After birth, the sphenoid sinus exists primarily as a pit in the sphenoethmoid recess; by age 3, starts pneumatization of the sphenoid bone; by age 7, extended toward the sella turcica; and it reaches the final form in the mid-teens. Although the definitive cavity forms at puberty, the actual sinus cavity starts becoming visible between 8–10 years. On the posterior nasal wall, the origin of the sphenoid sinus can be clearly identified by the location of its ostium [8]. The paired sinuses generally develop asymmetrically, separated by the inter-sinus bony septum [7,9]. During childhood, maturation of the bone from red to yellow marrow takes place in the anterior part of the sphenoid bone [10]. 

The sphenoid sinus is considered the most variable of the paranasal sinuses in terms of degree and type of pneumatization, number and position of inter-sinus septa, relationship with the surrounding structures like the cranial nerves (CN II, III, IV, V, VI), the internal carotid artery inside the cavernous sinus, and the pituitary gland [11,12]. The relationship of the sphenoid sinus is a prerequisite to safe and effective surgical treatment for lesions in the nasal region, and lack of orientation during dissection might lead to different surgical complications [8]. The sphenoid sinus drains directly into the nasal cavity by the sphenoethmoid recess [13]. Knowledge of anatomographic variations of the sphenoid sinus and its anatomic relationship with surrounding structures shorten the duration of surgeries while reducing further comorbid complications. Radiological evaluation of anatomographical variations of sphenoid sinus assist to understand the key steps taken during tumor progression as well as guide surgeons in the management of the relevant pathologies [14]. 

## 2. Materials and Methods

A prospective observational study was conducted at the Tikur Anbessa Specialized Teaching Hospital, Addis Ababa University, on 200 patients (117 female and 83 male) who underwent CT scan imaging for paranasal sinuses evaluation during 2017–2018. Ethical clearance was obtained from both, Department of Anatomy and Radiology Research Ethics Review Committee, DRERC/01/09, 06/01/2017. CT scan images were acquired from patients with informed consent. Data were anonymous to maintain patient confidentiality. Images with a slice thickness > 3 mm, low resolution quality, and those with metallic artifacts that impair sinus visualization were excluded from the study. In addition, patients with a history of prior sinus or sphenoid surgery, facial trauma, and obscured sphenoid sinus pathology were also excluded. A Philips 128 slice MDCT scanner, 130 kV, 120 mAs, was used to acquire images. Images were taken in the axial planes and then 1.25 mm slices were reconfigured into coronal and sagittal planes [15]. Patients were placed in the supine position with their chin hyperextended and scan plane angled perpendicular to the hard palate. Axial scans were performed from the maxillary sinus floor to the level of the frontal sinus roof, in a plane parallel with the hard palate [10]. Images were reviewed on the console with varying window levels and widths. The sphenoid sinuses were reviewed in both axial and coronal planes, and the total number of septa were counted (single, double, and absent) and compared in both planes [16]. The data was processed by Maxi and RadiAnt viewer computer softwares (Mission Viejo, CA 92691, USA and 5.5.1.23267, Medixant, Pozan’, Poland respectively). Selected images for the study were reviewed and interpreted by one neuroradiologist and five senior radiology residents. For detailed anatomographic variations, only findings consistently reported by all were used. The quantitative data was captured by an Excel spreadsheet, cleaned, and exported to SPSS version 20 for analysis. The mean, median, frequency distribution and proportion of variables indicating variation were reported using descriptive statistics. A *p*-value < 0.05 was considered statically significant based on the Chi-square tests.

## 3. Results

**Figure 1:**
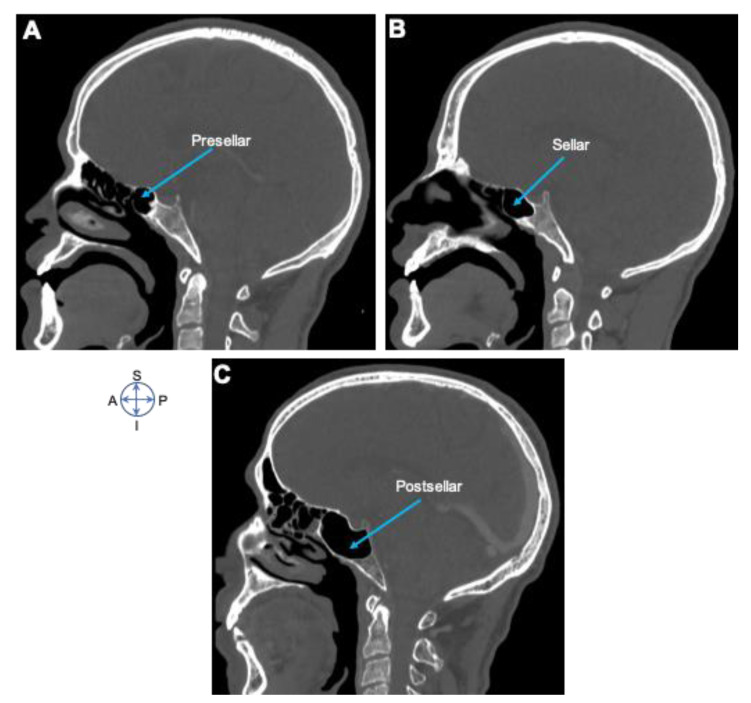
Anatomographic variants of sphenoid sinus. Sagittal CT images showing (**A**) presellar, (**B**) sellar, and (**C**) postsellar types of sphenoid sinus. Note that the sellar type of pneumatization was found to be the most frequent anatomographic variant of sphenoid sinus in the Ethiopian population. Image orientation: S, superior; I, inferior; A, anterior; P, posterior.

**Figure 2:**
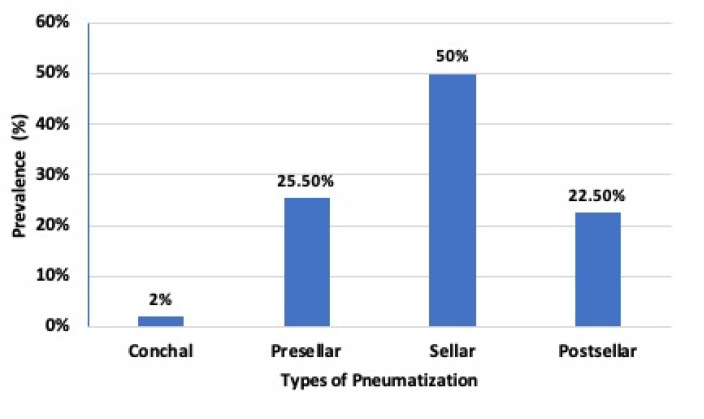
Prevalence of type of sphenoid sinus pneumatization.

**Figure 3:**
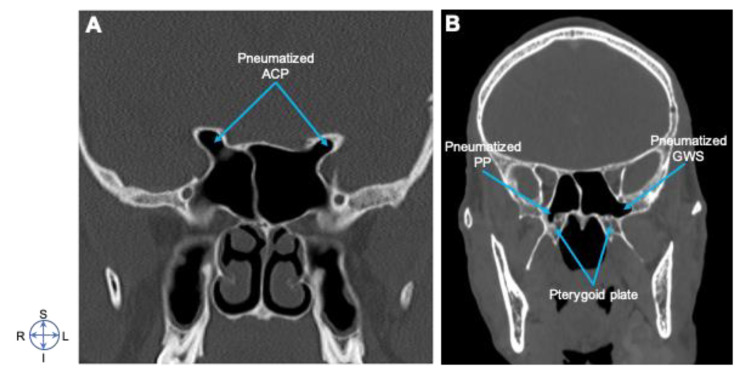
Pneumatization of the sphenoid sinus. Coronal CT images showing (**A**) Pneumatized anterior clinoid process (ACP); (**B**) Pneumatized pterygoid plate (PP) and greater wing of sphenoid (GWS). Note that half of the patient population participating in the study possessed sellar type of sphenoid sinus pneumatization. Image orientation: S, superior; I, inferior; R, right side; L, left side.

In this study, 200 patients participated, out of which 117 (58.5%) were females, 83 (41.5%) were males, and seven (3.4%) were non-respondents. The participants’ age ranged from 18 to 79, with a mean of (±SD) 43 (±14.5) years. The mean age of males was 51, while for females was 37 (Table 1). Our findings showed that anatomographic variants of sphenoid sinus were 100 (50%) seller, 51 (25.5%) presellar, 45 (22.5%) postsellar, and 4 (2%) were conchal types of pneumatization (Figure 1 and Figure 2; Table 2). We also found that pneumatization of the anterior clinoid process (ACP) was 36 (18%), pterygoid plates (PP) was 3 (15%), and the greater wing of sphenoid was (GWS) 33 (16.5%) (Figure 3).

**Figure 4:**
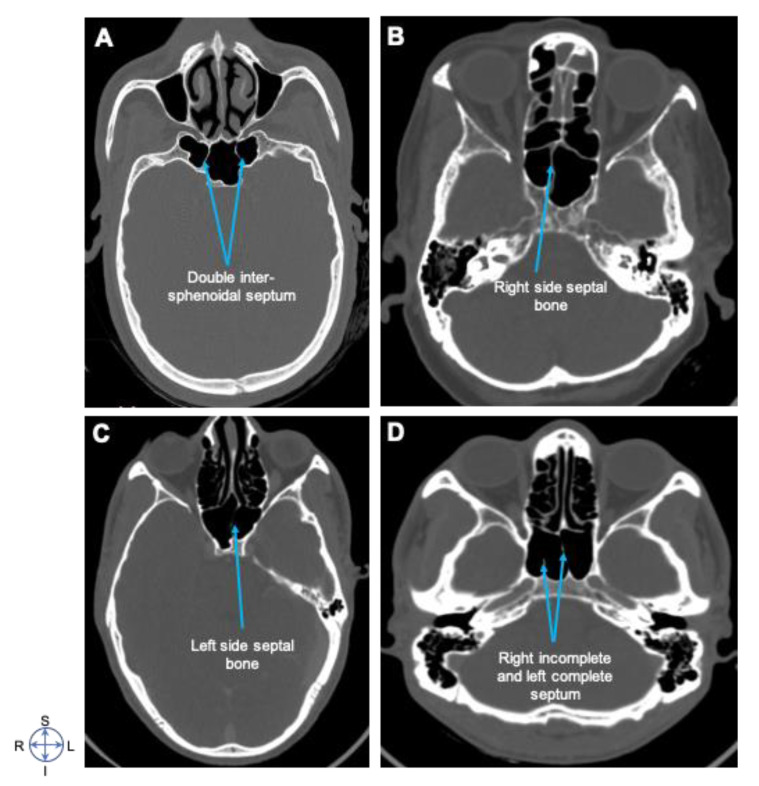
Septation of the sphenoid sinus. Axial CT images showing (**A**) Double inter-sphenoidal septum, (**B**) Right side septal bone, (**C**) Left side septal bone, and (**D**) Right incomplete and left complete septum. Note that almost 90% of the patient population who participated in this study showed single septation, whereas 1% without septation. Image orientation: S, superior; I, inferior; R, right side; L, left side.

**Figure 5:**
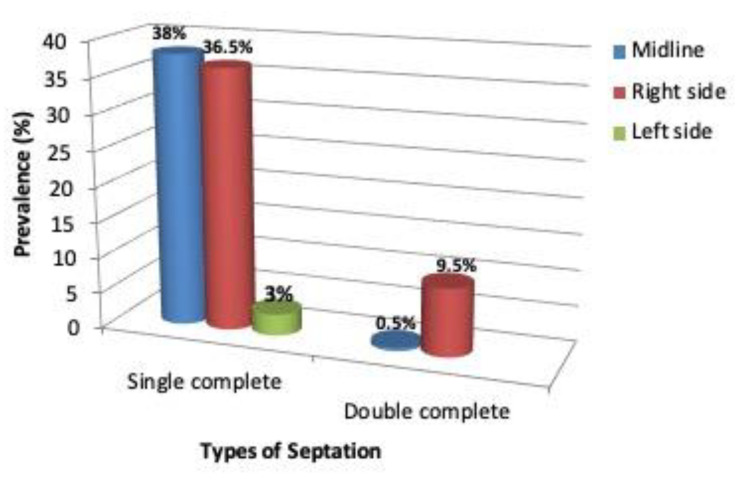
Sphenoid sinus septum in midline, right and left side orientation.

Septal bone septation of the sphenoid sinus in the Ethiopian population was single 179 (89%). That is, 115 (77.5%) complete and 23 (11.5%) incomplete), 20 (10%) double (all are complete), and two (1%) were either null or without septation (Figure 4 and Figure 5; Table 2). 

**Figure 6:**
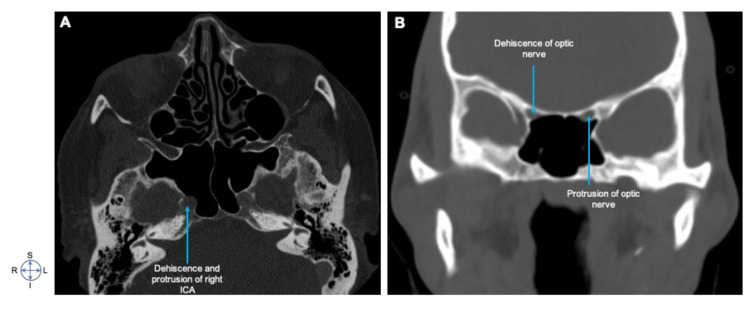
Dehiscence and protrusion of sphenoid sinus in relationship to internal carotid artery (ICA) and optic nerve. Axial CT image (**A**) showing dehiscence and protrusion of right ICA, (**B**) showing dehiscence of optic nerve (R) and protrusion of optic nerve (L). Note the differential protrusion and dehiscence in Ethiopian population. Image orientation: S, superior; I, inferior; R, right side; L, left side.

We also investigated the dehiscence and protrusion of sphenoid sinus in relation to internal carotid artery, optic nerve, and foramen rotundum (V2) (Table 3). Protrusion of the internal carotid artery into the sphenoid sinus was identified on the CT images of 37 (18.5%) patients: the right side alone was involved in 12 (32.4%) patients; the left alone in nine (24.3%) patients, and bilateral involvement was involved in 16 (43.2%) patients. The dehiscence of the bony sphenoidal wall of the internal carotid artery occurred in 24 (12%) patients; the right side alone was involved in seven (29.17%) patients, left side alone in 10 (41.7%) patients, and bilateral involvement was observed in seven (29.17%) patients. Nineteen (9.5%) cases had the optic nerve protrusion into the sphenoid sinus: right sided in one (5.26%) case, left side in 10 (52.63%) cases, and bilateral involvement in eight (42.1%) cases. However, dehiscence occurred in 31 (15.5%) patients; right sided in 12 (38.7%) cases, left side in 9 (29%) cases, and bilateral involvement in 10 (32.5%) cases. From the total participants, 25 (12.5%) cases had maxillary nerve protrusion into the sphenoid sinus; the right side alone was involved in seven (28%) patients; the left alone in eight (32%) patients, and bilateral involvement was involved in 10 (40%) patients. However, dehiscence occurred in 25 (12.5%) patients; right sided in four (16%) cases, left side in 11 (44%) cases, and bilateral involvement in 10 (40%) cases. We also identified the presence of onodi cells only in two (1%) cases (Figure 6). The Pearson Chi-square-χ^2^ test for the pterygoid plate and vidian canal dehiscence, dehiscence of the optic nerve, and pneumatization of the anterior clinoid process at the 95% confidence interval was *p* = 0.05, whereas protrusion of OPN and pneumatization of ACP were found to be statistically non-significant among this study population (Table 4).

## 4. Discussion 

Anatomical variations of the sphenoid sinus is well documented among many African populations [5,17], however, information is scanty among the Horn of African nations. Among them, Ethiopia is often considered as the cradle of mankind, the land of origins. Understanding anatomographic variants of the sphenoid sinus among these populations is of paramount importance to compare the variability or racial differences among black populations [18]. Paranasal sinus lesions are very common and affect a wide range of population with a variety of etiologies from inflammation to neoplasm [19]. Disease of the sphenoid sinus is difficult to diagnose and treat due to the fact that initial symptoms are vague to recognize. In addition to this, the rarity of sphenoid sinus involvement can be explained by the nonspecific symptoms, the inaccessibility to the sinus through the otorhinolaryngological physical examination, and the low number of diagnoses prior to the advent of more sophisticated technologies such as CT and magnetic resonance imaging [20].

Higher incidence of anatomographical variations of sphenoid sinus can lead to increased risk in terms of injury of important neurovascular and glandular structures [14,21]. Extensive hyperpneumatization of the sphenoid sinus with consecutive pneumatization of ethmoid sinus can lead to injury of the optic nerve [22]. Protrusion of the internal carotid artery into the lumen of the sinuses can also lead to its injury during endoscopic surgical procedures, especially in cases of variation in positions, numbers, and insertions within the sinus septum [23,24].

In this study, we pointed out that compared with presellar and postsellar, sellar was the most frequent anatomographic variant of sphenoid sinus in Ethiopian population (Figure 1 and Figure 2; Table 2). Our finding also indicated that 50% of the Ethiopians participating in the study possess the sellar type of sphenoid sinus pneumatization (Figure 3, Table 2). This finding is in line with the previous study reports of 52.9% [12], but was less than that of 59.4% [9] and 69% [25]. This indicates possible existence of racial anatomographic variants of the sphenoid sinus across different populations. Our findings showed presellar pneumatization in almost a quarter of the 200 study cases (25.5%), where 16.5% patients were on the left side, 6% patients were on the right, and 3% patients were bilateral. The conchal type of pneumatization was the least frequent (2%) in our study, where as in other studies it was 1.8% [2,19], 1.9% [12], and 3% [25], and these differences might be attributed to the variations in sample size selection in various studies. Our findings of pneumatization of ACP 18%, PP 15%, and GWS 16.5% was consistent with previous studies done at “Ovidius’’ University of Constanta, New Jersey, USA, and Oyo-State, Nigeria [4,19,25,26]. Furthermore, a study done in Constanta Romania found that 33% of the cases showed extensive pneumatization of the pterygoid process; in 10% of the cases, the anterior clinoid processes were pneumatized, forming deep optico-carotid recesses; and in 8% of the cases, there were lateral extensions of pneumatization in the greater wing of the sphenoid bone [25].

Sphenoid sinus septal bone variations are also considered the potential anatomographical variations illustrated in this region [27]. Our study elucidated that almost 90% of Ethiopian population participating in the study had single septation, whereas 1% were without septation. Out of the single septated cases, 38% were midline, 36.5% right side, and 3% on the left side, whereas single incomplete septa and double septa corresponded with each other at 11.5%. However, the absence of septa was observed only in 1% of the study population ((Figure 4 and Figure 5; Table 2)). Regarding the degree of septation, a single inter-sphenoidal septum was observed in 28.1%, and more than one septum in 71.9%. When this finding is compared with other study populations, it is seemingly higher than other studies done in Kerala, India, Turkey, and South Africa populations [7,28,29,30]. These results were different from other findings, possibly due to the presence of racial or anatomographical variants across different geographical or ancestral origins that might show the evolution of anatomical variations along ethno-geographical or racial diversity. In our study, the absence of septa was found in only 1% of the patients, while it was 2.7% in Nigerians [4,31] and 2.2% in other studies [32,33,34]. These findings are almost comparable despite the difference in their sample size. Corroborated with our finding, they all point to the rare occurrence of sphenoid sinus without septation in less than 2% across the different studied populations.

Our investigation on the relationship of the sphenoid sinus with ICA, OPN, and V2 are summarized in Figure 6 and Table 3. Protrusion and dehiscence of the sphenoid sinus with ICA was 18.5% and 12%, with OPN of 9.5% and 15.5%, and with V2 of 12.5% and 12.5%, respectively. This entails radiological evaluation that showed an equivalent protrusion and dehiscence of V2, higher protrusion of ICA, and higher dehiscence of OPN among the Ethiopians participated in the study, which is a clinically relevant anatomographic variant to be considered during surgical interventions involving the sphenoid sinus. These findings are also consistent with other findings [23,31].

In summary, the sellar type of sphenoid sinus pneumatization was found to be the most clinically relevant anatomographic variant among the Ethiopian population. Ninety percent of the patient population participating in the study were found to be tomographically single septated. A radiologically equivalent protrusion and dehiscence of the sphenoid sinuses can be considered as anatomographical variations during neurosurgical interventions in the Ethiopian population. The vast majority of this variability is attributed to the extent of sphenoid sinus pneumatization, varying number and position of septae, and the anatomical relationship with surrounding neurovascular and glandular structures visible during imaging modalities. To the best of our knowledge, this is the first diagnostic report in Eastern Africa incorporating both anatomical and radiological data. We also recommend further investigation in the populations of common ancestral origins.

## Figures and Tables

**Table 1:** Age distribution of patients who participated in the study (*n* = 200).

Age in Years	Minimum	Mean	Maximum
Male	18	51	79
Female	18	37	74
Both	18	43	79

**Table 2:** Incidence of sphenoid sinus pneumatization and septation in the Ethiopian population.

Anatomographic Variants of the Sphenoid Sinus					
Pneumatization		Septation			
Type	Frequency	Type	Midline	Right Side	Left Side
Conchal	4 (2%)	Single complete	76 (38%)	73 (36.5%)	6 (3%)
Presellar	51 (25.5%)	Double complete	1 (0.5%)	19 (9.5%)	0 (0%)
Sellar	100 (50%)	No septa	2(1%)	0 (0%)	0 (0%)
Postsellar	45 (22.5%)				

**Table 3:** Summary of relationship of internal carotid artery, optic nerve and foramen rotundum to the sphenoid sinus.

		Sides			
Structure		Right Side	Left Side	Bilateral	Total
ICA *	Protrusion	12 (32.4%)	9 (24.3%)	16 (43.2%)	37 (18.5%)
	Dehiscence	7 (29.17%)	10 (41.7%)	7 (29.17%)	24 (12%)
OPN *	Protrusion	1 (5.26%)	10 (52.63%)	8 (42.1%)	19 (9.5%)
	Dehiscence	12 (38.7%)	9 (29%)	10 (32.25%)	31 (15.5%)
V2 *	Protrusion	7 (28%)	8 (32%)	10 (40%)	25 (12.5%)
	Dehiscence	4 (16%)	11 (44%)	10 (40%)	25 (12.5%)

* ICA (internal carotid artery); OPN (optic nerve); V2 (foramen rotundum).

**Table 4:** Chi-square Tests.

(df = 1)	Pearson Chi-Square-χ^2^	*p* 95% Confidence Interval
Dehiscence of VDC * pneumatization PP *	7.864	0.049
Protrusion of OPN * pneumatization of ACP *	0.584	0.747
Dehiscence of OPN * pneumatization of ACP *	7.945	0.046

* VDC, Vidian Canal; PP, Pterygoid Plate; OPN, Optic Nerve; ACP, Anterior Clinoid Process. (df = 1) Pearson Chi-square-χ^2^ *p* 95% confidence interval pterygoid plate, optic nerve and anterior clinoid process.
